# Myokines in Acromegaly: An Altered Irisin Profile

**DOI:** 10.3389/fendo.2021.728734

**Published:** 2021-11-02

**Authors:** Łukasz Mizera, Jowita Halupczok-Żyła, Katarzyna Kolačkov, Agnieszka Zembska, Jędrzej Grzegrzółka, Diana Jędrzejuk, Marek Bolanowski, Jacek Daroszewski

**Affiliations:** ^1^ Department of Endocrinology, Diabetes and Isotope Therapy, Wrocław Medical University, Wrocław, Poland; ^2^ Department of Histology and Embryology, Wrocław Medical University, Wrocław, Poland

**Keywords:** irisin, myostatin, acromegaly, GH, IGF-1, muscles, myokines

## Abstract

**Introduction:**

The muscle is an endocrine organ controlling metabolic homeostasis. Irisin and myostatin are key myokines mediating this process. Acromegaly is a chronic disease with a wide spectrum of complications, including metabolic disturbances.

**Purpose:**

To examine the influence of acromegaly on irisin and myostatin secretion and their contribution to metabolic profile and body composition.

**Materials and Methods:**

In 43 patients with acromegaly and 60 controls, serum levels of irisin, myostatin, growth hormone (GH), insulin-like growth factor 1 (IGF-1), parameters of glucose, and lipid metabolism were determined. Body composition was assessed with dual-energy x-ray absorptiometry.

**Results:**

The irisin concentration was significantly lower in patients with acromegaly compared to controls (3.91 *vs*. 5.09 μg/ml, *p* = 0.006). There were no correlations between irisin and GH/IGF-1 levels. In the study group, irisin was negatively correlated with fasting insulin (*r* = −0.367; *p* = 0.042), HOMA-IR (*r* = −0.510; *p* = 0.011), and atherogenic factors: Castelli I (*r* = −0.416; *p* = 0.005), Castelli II (*r* = −0.400; *p* = 0.001), and atherogenic coefficient (AC) (*r* = −0.417; *p* = 0.05). Irisin and myostatin concentrations were also lower in acromegalics with insulin resistance than without (2.80 *vs*. 4.18 μg/ml, *p* = 0.047; 81.46 *vs*. 429.58 ng/L, *p* = 0.018, respectively). There were no differences between study group and controls in myostatin concentration. Myostatin levels negatively correlated with GH (*r* = −0.306; *p* = 0.049), HOMA-IR (*r* = −0.046; *p* = 0.411), and insulin levels (*r* = −0.429; *p* = 0.016).

**Conclusions:**

Decreased irisin concentrations in acromegaly may suggest impaired hormonal muscle function contributing to metabolic complications in this disorder. However, learning more about the association between myostatin and GH in acromegaly requires further studies. Nevertheless, it appears that myostatin is not critical for muscle mass regulation in acromegaly.

## Introduction

Currently, the skeletal muscle role is considered to extend far beyond its mechanical function. Studies of the last two decades identified the muscle as the largest endocrine organ. Its secretome consists of numerous peptides regulating multiple physiological processes in an auto-, para-, and endocrine manner, especially muscle growth and energy metabolism, but also immune, endothelial, and central nervous system function (1).

In 2012, Bostrom et al. discovered a new myokine—irisin, whose activity can counteract metabolic disorders in a remarkable way. Under the influence of irisin, white adipose tissue (WAT) acquires brown adipose tissue (BAT)-like properties through the formation of uncoupling protein 1 expressing beige adipocytes, which disperse energy in the process of non-shivering thermogenesis. In this regard, beige adipocytes utilize glucose and free fatty acids in a non-insulin-dependent way (2), which protects against lipotoxicity and favors weight loss (3). Recent studies have shed light on the pleiotropic action of irisin. Through interaction with its receptor in various cells, this myokine exhibits a profound effect on glucose and lipid metabolism in a manner non-dependent on WAT browning. Irisin was found to increase muscle and liver insulin sensitivity (4, 5), act as a β-cell secretagogue, promote β-cell proliferation and survival in gluco- and lipotoxic conditions (6–8), as well as aid to maintain metabolic homeostasis through increasing autophagy (9, 10).

Myostatin is a member of the transforming growth factor β (TGF-β) superfamily protein abundantly expressed and secreted from muscle. Furthermore, myostatin is a potent negative regulator of muscle growth and development. It acts by inhibiting myogenic stem cells and myocytes, preventing hyperplasia and hypertrophy (11). Deletion of the myostatin gene results in the famous double-muscle phenotype in cattle (12). This type of mutation was also described in humans yielding extreme muscle hypertrophy (13). Besides its influence on the skeletal muscle, myostatin is also highly involved in the regulating of glucose metabolism. Myostatin antagonizes insulin action on the post-receptor phase through inhibition of Akt phosphorylation (14, 15) and inhibitory effect on irisin secretion (16). In consequence, a depressed function of this myokine results in a better metabolic phenotype with resistance to high-fat diet-induced obesity and insulin resistance (11, 17). Conversely, the administration of recombinant myostatin induces insulin resistance (14).

Acromegaly is a chronic systemic disease, most often caused by a benign somatotroph pituitary adenoma and may be considered a natural model of chronic GH excess. Metabolic complications are common in this disease and contribute to a 1.5–2.5 times higher standardized mortality ratio with cardiovascular disease as a major cause of death (18, 19). GH antagonizes insulin action and increases lipolysis causing insulin resistance and lipotoxicity (20–23). High GH levels also promote altered adipose tissue distribution and phenotype with unfavorable adipokine profiles (24). On the other hand, IGF-1 may have some beneficial effects with its weak insulin activity (25) and possible ability to reduce low-density lipoprotein (LDL) oxidation (26), and GH stimulates β-cell proliferation and function. In the case of active acromegaly, most patients are insulin resistant, and the rates of overt glucose abnormalities are increased several times compared to the general population (27, 28). Insulin resistance is a factor strongly predisposing to cardiovascular complications, hypertension, diabetes, obstructive sleep apnea, and reproductive health issues, and influences cancer promotion (29). Lipid abnormalities affect 13%–53% of acromegaly patients, mainly manifesting as atherogenic dyslipidemia. The occurrence of hypercholesterolemia in acromegaly is less well-documented, with some studies suggesting higher and some normal levels (30). The successful treatment causes improvement in metabolic disorders; however, the increased risk of glucose abnormalities persists (31). Given its pathophysiological background, patients with acromegaly at different stages of treatment are a suitable model for studies of growth hormone activity. Thus, we performed a study assessing myokine profile in these patients to investigate the effect of GH overproduction on muscle secretome in the context of myostatin and irisin secretion. In addition, we examined the potential influence of irisin and myostatin concentrations on metabolic complications in this disease.

## Materials and Methods

### Patients

In this single-center cross-sectional study, 43 patients with acromegaly constituting the study group and 60 controls were enrolled. The study was approved by the bioethics committee of the Wrocław Medical University.

Diagnosis of acromegaly was compliant with international guidelines (32). Detailed patient data are presented in **Table 1**. In the study group, 38 patients underwent surgery—among those, 15 were without pharmacotherapy and 25 patients received somatostatin analogs (SSA). In addition, the patients were not treated with pegvisomant or cabergoline and five patients had radiotherapy. The median time from diagnosis was 6 years (range 0–34 years). Among patients with acromegaly, 12 patients had the active and 31 controlled form of the disease. Patients were considered controlled if they showed GH suppression below 1 ng/ml in an oral glucose tolerance test (OGTT) and IGF-1 within the normal range for age and sex in patients treated only surgically or random GH was <2.5 ng/ml with IGF-1 within the normal range for age and sex in patients treated pharmacologically.

**Table 1:** Clinical characteristics of the study group and controls.

	Acromegaly	Controls	*p*
Age (years)	67.72 ± 11.44	58.25 ± 12.56	0.531
Sex (men/women)	15/28	21/39	0.840
IR (+/-)	10/14	16/27	0.720
Prediabetes (+/-)	7/20	11/40	0.664
Diabetes (+/-)	11/32	10/50	0.268
Hypertension (+/-)	28/15	29/28	0.154
Atherogenic dyslipidemia (+/-)	6/37	6/51	0.602
Hypercholesterolemia (+/-)	22/21	22/35	0.210
Cardiovascular disease (+/-)	3/38	8/47	0.271

The control group consisted of patients without endocrinopathies, with the exception of diabetes and any known inflammatory disease. Controls were matched to the study group in terms of age, sex, and the occurrence of diabetes and lipid abnormalities.

For analytical purposes, the study group was further subdivided according to:

- Insulin resistance (IR)—patients were assigned to the IR (+) group if diabetes was excluded and their HOMA-IR exceeded 2.1. The cutoff was chosen based on the upper quartile determined for the Polish population (33).
- Diabetes and prediabetes (defined as impaired fasting glucose [IFG] or impaired glucose tolerance [IGT])—patients were assigned to the diabetes (+) or prediabetes (+) group according to known pre-existing diabetes or based on fasting glucose level and 75 g oral glucose tolerance test using criteria proposed by the American Diabetes Association (34).
- Atherogenic dyslipidemia—patients were assigned to atherogenic dyslipidemia group (+) if the following criteria was met: TG >150 mg/dl and HDL-C <40 mg/dl for men and <45 mg/dl for women (35).
- Hypercholesterolemia—patients were assigned to the hypercholesterolemia (+) group if their total cholesterol (TC) or low-density lipoprotein cholesterol (LDL-C) exceeded the upper limit of the norm for the assay or were treated for hypercholesterolemia

### Methods

#### Biochemical Analyses

All fasting blood samples were collected, after at least 1 h of rest. Serum aliquots were stored at −70°C. Irisin was measured by ELISA assay (Biovendor—Laboratorni medicina a.s., Czech Republic, range 0.001–5 µg/ml, sensitivity 1 ng/ml). Myostatin was measured by ELISA assay (Bioassay Technology Laboratory, Shanghai, China, range 0.5–2000 ng/L, sensitivity 0.25 ng/L). All reactions were run in duplicate.

Glucose and lipids were measured by immunochemiluminometric methods (ARCHITECT, Abbott Diagnostics). LDL-C levels were measured using the Friedewald formula (36). Serum insulin, GH, and IGF-1 were measured by immunochemiluminometric assay (IMMULITE 2000, Siemens Healthcare Diagnostics).

#### Insulin Resistance, β-Cell Function Indices, and Atherogenic Factors

Insulin resistance and β-cell function indices were calculated only for subjects without diabetes. Homeostasis model assessment for insulin resistance for β-cell function (HOMA-β) was calculated using the following formula: 20 × fasting insulin (μIU/ml)/fasting glucose(mmol/ml) − 3.5. Homeostasis model assessment for insulin resistance (HOMA-IR) was calculated using the following formula: fasting insulin [μIU/ml] × fasting glucose [mg/dl]/18/22.5.

#### Atherogenic Factors

For each patient, the following five atherogenic factors were calculated:

- Atherogenic Index of Plasma (AIP) (37);
- Castelli I (37);
- Castelli II (37);
- Atherogenic coefficient (AC) (37); and
- TG/high-density lipoprotein (HDL) ratio (38).

#### Body Composition Measures

Body composition measures were assessed by dual-energy x-ray absorptiometry (DXA) using a Hologic Discovery QDR Series densitometer (Hologic Incorp. USA, APEX 4.5.2.1 software version, Windows 7 Professional system). The following body composition measures were assessed:

- Total body fat percentage (% fat);
- Fat mass (FM);
- Fat mass index (FMI);
- Fat free mass index (FFMI);
- Lean mass (LM);
- Lean mass index (LMI);
- Appendicular lean mass index (ALMI);
- Appendicular lean mass/BMI (ALM/BMI); and
- Trunk body fat percentage/legs body fat percentage—reflecting the visceral to subcutaneous fat ratio (% fat trunk/% fat legs).

### Statistical Analysis

The Kolmogorov–Smirnov test was performed to assess the normality assumption. The number of cases in each category was compared using the Chi-square or Fisher exact test. The unpaired *t*-test or Mann–Whitney test was used to compare the differences of the parameters in the examined groups. Comparison between more than two groups was made by the ANOVA or Kruskal–Wallis and Dunn’s multiple comparison test. Finally, the Pearson or Spearman correlation test was used to examine the existing correlations. All statistical analyses were performed using Prism 5.0 (GraphPad, La Jolla, California, USA) and STATISTICA 10 (StatSoft Inc. Tulsa, Oklahoma, USA). The significance threshold was set at *p* < 0.05.

## Results

### Comparison of Clinical and Laboratory Characteristics

There were no significant differences between the study group and controls in age, sex, insulin resistance, prediabetes, diabetes, hypertension, lipid disturbances, and known cardiovascular disease (**Table 1**).

Patients with acromegaly demonstrated significantly lower serum irisin concentrations than controls (3.91 *vs*. 5.09 μg/ml, *p* = 0.006) (**Table 2**). Myostatin concentrations did not differ among the studied groups.

**Table 2:** Laboratory parameters in patients with acromegaly and controls.

	Acromegaly	Controls	*p*
Irisin (μg/ml)	3.91 (2.08–19.60)	5.09 (2.50–12.70)	0.006*
Myostatin (ng/L)	91.75 (56.90–1766.40)	348.53 (59.62–1619.32)	0.220
GH (ng/ml)	1.32 (0.05–29.50)	0.25 (0.05–5.54)	<0.001*
IGF-1 (ng/ml)	182.17 ± 115.62	112.5 ± 42.27	<0.001*
Glucose (mg/dl)	97.63 ± 13.94	92.62 ± 14.42	0.093
HbA1c (%)	6.10 (5.10–9.50)	5.5 (4.70–12.40)	0.001*
Insulin (μIU/ml)	7.81 (2.00–38.30)	7.99 (2.00–30.50)	0.477
HOMA-IR	1.68 (0.37–4.74)	1.67 (0.42–8.59)	0.561
HOMA-β	94.75 ± 54.15	139.18 ± 92.60	0.035*
Total cholesterol (mg/dl)	188.17 ± 47.13	201.04 ± 40.21	0.141
LDL-C (mg/dl)	114.95 ± 39.27	123.93 ± 35.63	0.246
HDL-C (mg/dl)	49.00 (31.00–84.00)	54.00 (28.00–158.00)	0.293
TG (mg/dl)	107.91 ± 47.40	118.73 ± 43.79	0.245
AIP	0.29 ± 0.27	0.33 ± 0.25	0.494
Castelli I	3.77 ± 0.99	3.89 ± 1.06	0.550
Castelli II	2.28 ± 0.85	2.4 ± 0.90	0.504
AC	2.77 ± 0.99	2.89 ± 1.06	0.550
TG/HDL	2.5 ± 1.45	2.43 ± 1.40	0.776
BMI (kg/m^2^)	29.75 ± 5.36	27.57 ± 5.31	0.046*
FM (kg)	29.31 ± 8.02	27.72 ± 9.95	0.401
LM (kg)	51.43 ± 11.20	46.28 ± 10.19	0.022*
%fat (%)	35.26 ± 7.65	36.00 ± 7.68	0.642
FMI (kg/m^2^)	10.46 ± 3.20	10.03 ± 3.61	0.545
FFMI (kg/m^2^)	18.78 ± 2.55	17.24 ± 2.52	0.004*
LMI (kg/m^2^)	17.92 ± 2.49	16.45 ± 2.44	0.005*
ALMI (kg/m^2^)	8.00 ± 1.31	7.66 ± 1.40	0.109
ALM/BMI	0.72 ± 0.19	0.74 ± 0.18	0.682

Results are presented as mean ± SD or median (minimum–maximum). LDL-C, LDL cholesterol; HDL-C, HDL cholesterol; TG, triglyceride; AIP, atherogenic index of plasma; AC, atherogenic coefficient; FM, fat mass; LM, lean mass; %fat, whole-body fat percentage; FMI, fat mass index; FFMI, fat free mass index; LMI, lean mass index; ALMI, appendicular lean mass index; ALM/BMI, appendicular lean mass to BMI ratio. *Statistically significant (p<0.05).

Serum GH and IGF-1 levels were significantly higher in patients with acromegaly compared to controls (0.25 ng/ml *vs*. 1.32 ng/ml and 112.5 ng/ml *vs*. 182.17 ng/ml, respectively, *p* < 0.001) (**Table 2**). BMI, LM, FFMI, and LMI were higher in acromegaly patients than in controls. The HbA1c percentage was higher in the study group than controls (6.1% *vs*. 5.5%, *p* = 0.001), whereas HOMA-β was significantly lower in the study group than controls (94.75 *vs*. 139.18, *p* = 0.035) (**Table 2**).

Comparison of the patients with active acromegaly, controlled disease, and controls did not reveal significant differences in irisin and myostatin serum concentrations (**Table 3**).

**Table 3:** Circulating myokine concentrations in patients with active acromegaly, controlled acromegaly, and controls.

	Active acromegaly	Controlled acromegaly	Controls	*p*
Irisin (μg/ml)	4.59 ± 2.32	4.75 ± 3.66	5.28 ± 1.97	0.502
Myostatin (ng/L)	105.20 (60.06–1,467.04)	91.56 (56.92–1,766.41)	348.53 (59.62–1,619.32)	0.394

In the subgroup analyses of patients with acromegaly, those with IR had significantly lower serum irisin (2.80 *vs*. 4.18 μg/ml, *p* = 0.047) and myostatin (81.46 *vs*. 429.58 ng/L, *p* = 0.018) concentrations than those without IR (**Table 4**).

**Table 4:** Circulating myokine concentrations in acromegaly patients with and without selected complications.

	Complication present (+)		Complication absent (-)		*p*		
	Irisin (μg/ml)	Myostatin (ng/L)	Irisin (μg/ml)	Myostatin (ng/L)	Irisin		Myostatin
IR	2.80 (2.15–19.60)	81.46 (56.92–269.21)	4.18 (2.40–10.95)	429.58 (74.02–1,467.04)	0.047		0.018*
Prediabetes	2.82 (2.44–4.08)	91.75 (56.92–1,315.92)	4.25 (2.08–19.60)	227.83 (60.06–1,766.41)	0.128		0.847
Diabetes	4.88 ± 3.51	137.14 ± 83.51	4.62 ± 1.76	413.92 ± 516.62	0.816		0.09
Hypercholesterolemia	3.42 (2.13–10.95)	134.65 (56.92–1,766.41)	4.43 (2.08–19.60)	88.28 (61.36–1,421.98)	0.444		0.325
Atherogenic dyslipidemia	3.18 (2.44–5.70)	105.20 (69.26–865.09)	4.03 (2.08–19.60)	91.56 (56.92–1,766.41)	0.483		0.986

*Statistically significant (p<0.05).

The circulating levels of irisin and myostatin did not significantly differ among subgroups of patients with acromegaly divided according to prediabetes, diabetes, hypercholesterolemia, and atherogenic dyslipidemia (**Table 4**).

### Correlations

In patients with acromegaly, irisin was negatively correlated with fasting insulin (*r* = −0.367; *p* = 0.042), HOMA-IR (*r* = −0.510; *p* = 0.011), Castelli I (*r* = −0.416; *p* = 0.005), Castelli II (*r* = −0.400; *p* = 0.001), and AC (*r* = −0.417; *p* = 0.005) whereas in controls, irisin correlated significantly only with age (*r* = −0.429; *p* = 0.016) (**Table 5**).

**Table 5:** Correlations of the circulating myokine concentrations with metabolic parameters in patients with acromegaly.

	Acromegaly				Controls			
	Irisin		Myostatin		Irisin		Myostatin	
	*r*	*p*	*r*	*p*	*r*	*p*	*r*	*p*
Age (years)	0.082	0.603	−0.293	0.056	−0.429*	0.016*	0.162	0.250
GH (ng/ml)	0.083	0.599	−0.306*	0.049*	−0.044	0.842	0.273	0.786
IGF-1 (ng/ml)	−0.206	0.185	−0.209	0.177	0.217	0.320	0.032	0.974
Glucose (mg/dl)	−0.162	0.313	−0.297	0.059	0.165	0.171	0.045	0.752
HbA1c (%)	0.135	0.432	−0.202	0.236	0.284	0.080	0.076	0.650
Insulin (μIU/ml)	−0.367*	0.042*	−0.429*	0.016*	−0.191	0.382	−0.150	0.324
HOMA-IR	−0.510*	0.011*	−0.411*	0.046*	−0.194	0.374	−0.146	0.374
HOMA-β	−0.049	0.821	−0.371	0.074	−0.121	0.584	−0.118	0.752
Total cholesterol (mg/dl)	−0.187	0.229	0.203	0.192	0.393	0.064	0.033	0.818
LDL-C (mg/dl)	−0.283	0.076	0.243	0.130	0.291	0.177	0.042	0.769
HDL-C (mg/dl)	0.257	0.097	0.073	0.638	0.174	0.428	0.190	0.181
TG (mg/dl)	−0.102	0.515	−0.164	0.292	0.108	0.623	−0.152	0.292
AIP	−0.199	0.201	−0.134	0.392	−0.053	0.810	−0.160	0.273
Castelli I	−0.416*	0.005*	0.168	0.281	0.039	0.860	−0.163	0.257
Castelli II	−0.400*	0.010*	0.193	0.233	0.015	0.946	−0.105	0.462
AC	−0.417*	0.005*	0.168	0.281	0.039	0.860	−0.163	0.257
TG/HDL	−0.199	0.201	−0.134	0.392	−0.050	0.822	−0.184	0.201
BMI (kg/m^2^)	−0.060	0.705	−0.246	0.112	0.067	0.761	−0.277*	0.047*
FM (kg)	0.074	0.639	−0.176	0.264	0.140	0.525	−0.153	0.292
LM (kg)	−0.184	0.243	−0.106	0.503	−0.080	0.716	−0.325*	0.024*
%fat (%)	0.140	0.376	−0.025	0.875	0.133	0.546	0.104	0.477
FMI (kg/m^2^)	0.073	0.645	−0.143	0.366	0.150	0.493	−0.089	0.545
FFMI (kg/m^2^)	−0.118	0.458	−0.266	0.089	−0.065	0.767	−0.368*	0.01*
LMI (kg/m^2^)	−0.125	0.430	−0.267	0.087	−0.072	0.743	−0.367*	0.001*
ALMI (kg/m^2^)	−0.163	0.301	−0.191	0.226	−0.105	0.633	−0.390*	0.006*
ALM/BMI	−0.122	0.440	0.070	0.656	−0.090	0.683	−0.221	0.131

LDL-C, LDL cholesterol; HDL-C, HDL cholesterol; TG, triglyceride; AIP, atherogenic index of plasma; AC, atherogenic coefficient; FM, fat mass; LM, lean mass; %fat, whole-body fat percentage; FMI, fat mass index; FFMI, fat free mass index; LMI, lean mass index; ALMI, appendicular lean mass index; ALM/BMI, appendicular lean mass to BMI ratio. *Statistically significant (p<0.05).

In patients with acromegaly, myostatin was negatively correlated with GH, fasting insulin, and HOMA-IR. In controls, myostatin was negatively correlated with BMI (*r* = −0.277; *p* = 0.047), lean and muscle mass indices: LM (*r* = −0.325; *p* = 0.024), LMI (*r* = −0.367; *p* = 0.001), FFMI (*r* = −0.368; *p* = 0.01), and ALMI (*r* = −0.390; *p* = 0.006) (**Table 5**). We found no associations between the duration of the disease and irisin or myostatin levels.

## Discussion

Irisin and myostatin are crucial myokines participating in the regulation of metabolic homeostasis controlled by the muscle. Acromegaly is a systemic disorder with many complications, including metabolic disturbances. Therefore, we investigated the myokine secretion profile in patients with different activity of acromegaly.

### Irisin

For the first time, we showed that acromegaly is associated with decreased circulating irisin levels, which points toward the negative impact of chronic GH excess on irisin secretion. Nonetheless, this association does not appear to be connected with GH levels directly, as there was no statistically significant difference between active and controlled acromegaly groups, and irisin did not correlate with GH and IGF-1 levels. A decrease in irisin secretion seems to be a sustained effect of chronic supraphysiological GH levels, persistent even after achieving the normal hormonal status. This would not be surprising given the fact that the control of acromegaly in many cases causes an improvement rather than a full recovery from the disease complications (19, 31, 39, 40).

The factors associated with irisin levels in acromegaly patients were insulin resistance indices (HOMA-IR, fasting insulin) and atherogenic factors (Castelli I Castelli II, AC). Moreover, in the subgroup analysis, patients with acromegaly and IR had lower serum irisin concentration than those without IR. These findings likely reflect the beneficial effect of irisin on carbohydrate metabolism in this group of patients. The results in the study group differ from the controls, for whom irisin correlated only with age. Irisin is an insulin-sensitizing hormone, which has a positive effect on lipid profile through its pleiotropic action. Despite this, the majority of studies (41–44) including recent meta-analysis (45), but not all (46, 47), indicate that circulating irisin levels are positively associated with unfavorable metabolic parameters, including IR and lipid disorders in non-diabetic patients. These observations are commonly explained by the phenomenon of “irisin resistance”, which might contribute to developing metabolic disturbances or adaptive increased compensatory secretion to restore homeostasis. Thus, the negative correlation between IR indices and atherogenic factors in our study group was unexpected. These findings signal a different pathophysiological background of metabolic abnormalities in acromegaly than in the general population. Whereas in the latter, the presumed irisin resistance or compensatory increased secretion occurs, in acromegaly, there may be relative irisin deficiency with preserved normal irisin action reflected by lower serum concentration of this myokine and negative association with adverse metabolic parameters.

On the basis of the results of our study, we hypothesize that there is a long-lasting impairment in hormonal muscle function in conditions of long-term GH excess that contributes to the development of metabolic complications. Decreased irisin concentrations are associated with muscle pathology. Whereas acute muscle injury may cause increased irisin levels (48), several studies suggest a decrease in circulating irisin in chronic myopathies such as myotonic dystrophy (49), sarcopenia (50), and Cushing disease (51) or hypothyroidism (52)-associated myopathies. Until now, little attention has been paid to muscle disorders in acromegaly, but some research points toward the existence of myopathy connected with this disease, clinically manifesting as muscle weakness (53–55), diplopia (56), and muscle atrophy (55) with inhomogeneous pattern in muscle biopsy examination (57–59) and occasionally elevated creatine kinase or myopathic electromyography record (57, 60). Impairment in muscle hormonal function in acromegaly would be the other side of the coin of this complication. Together with the results of our study, this would imply the existence of acromegalic muscle disease with impaired mechanical and endocrine function. We postulate to introduce the term “acromegalic myopathy” with myokine metabolism dysfunction as a component. It is worth noting that many years ago, our team suggested that the activity of the cathepsin B enzyme, nowadays considered a myokine, may be taken into account as an overall adjuvant marker of the acromegaly complications (61).

Current knowledge about myokine profile in acromegaly is scarce, and circulating irisin levels in this disease were evaluated only in one study by Calan et al. (62). The authors found an increased irisin concentration in the active as well as controlled acromegaly as compared to the controls, positively associated with IR indices and non-classic cardiovascular disease risk factors. Although we cannot find definite reasons for these discrepancies, different populations with dissimilar treatment patterns, varying age, disease duration, physical activity status, and disease complication rates may contribute, particularly since, in the study of Calan et al., patients seem to be younger and have higher rates of diabetes, hypertension, and worse lipid measures, even in the controlled acromegaly group, than in our study. In this regard, it is worth noting that acromegaly was reported as an inhomogeneous disease with distinct pathogenesis, clinical picture, and more aggressive disease in young patients (63). Moreover, with the duration of the disease, its clinical picture may vary. Acromegalic cardiomyopathy may be a notable example when initially GH excess induces physiological cardiac muscle growth with functional benefit. Still, with time, fibrosis occurs, leading to diastolic dysfunction, valvular defects, and risk of arrhythmias, and consequently heart failure with impaired ejection fraction (64). Anyway, we must stress that our result strictly fits the contemporary mechanistic understanding of irisin function in metabolic disorders.

### Myostatin

To the best of our knowledge, this is the first study assessing myostatin levels in acromegaly. We found no significant differences in circulating myostatin levels between patients with acromegaly, neither active nor controlled, and controls. Nevertheless, further data analysis demonstrated that serum myostatin in the study group was negatively correlated with GH levels. This observation points towards negative regulation of myostatin levels by GH in acromegaly. The effect of GH on myostatin secretion is not clearly established in general. There are several *in vitro* studies that yield inconsistent results (65–67). Yet, Liu et al. demonstrated that patients with growth hormone deficiency (GHD) are characterized by elevated circulating myostatin levels and increased myostatin mRNA expression. Moreover, treatment with GH in these subjects resulted in a reduction of myostatin concentration and mRNA expression parallel to an increase in lean mass (68). Nonetheless, in another group of GHD patients, GH therapy did not affect myostatin gene expression. However, the conflicting findings may be connected with lower doses and shorter treatment time (6 *vs*. 18 months) (69). Indeed, the full effect of GH treatment on muscle tissue may require a longer time, exceeding 6 months (70). In mechanistic studies, GH increases whole-body protein synthesis without affecting proteolysis and induces muscle hypertrophy. The effect is mediated by IGF-1, but direct GH influence is also debated (71). Although administration of GH has a controversial effect on muscle tissue in healthy subjects, in patients with GHD, decrease in muscle mass and strength is well documented and improves after initiation of substitution therapy (72, 73). Possible negative regulation of myostatin by GH in acromegaly raises the question about its relevance in the anabolic effect of GH on muscle tissue. In the study by Liu et al., GH-induced decrease in myostatin was connected with an increase in lean mass (68). In another study, no impact on myostatin of the same treatment was paralleled by no effect on muscle mass measures (69). These observations suggest that treatment-induced muscle growth in GHD is dependent on myostatin inhibition, indeed, though, in our study, we did not observe any correlation of myostatin with any muscle mass indices. It should be noted that this observation differed from the control group in which myostatin was significantly correlated with ALMI, LM, LMI, and FFMI. The current study results indicate that in contrast to the general population, the role of myostatin is not critical for muscle mass regulation in acromegaly.

Serum myostatin concentration in the study group was negatively correlated with HOMA-IR and fasting insulin. Additionally, myostatin was significantly lower in the subgroup of acromegalics with IR compared to those without IR. Myostatin is more than a muscle mass regulator and has a well-established, powerful effect on glucose metabolism, mostly through insulin antagonism (14, 15). In overweight and obese subjects, muscle myostatin expression was found to be inversely correlated with insulin sensitivity (74). Moreover, after bariatric surgery or systematic aerobic activity improvement of insulin sensitivity, myostatin gene expression in muscle as well as muscle and serum myostatin concentrations decline (14, 75). An increase in myostatin muscle expression was reported in patients with diabetes, prediabetes, and in close relatives of diabetic patients (76–78). In the light of these observations and the fact that myostatin was negatively associated with GH, the discordant finding in our study is possibly a result of the joint influence of GH on myostatin and IR indices, given the diabetogenic action of GH. Therefore, a negative correlation of myostatin with HOMA-IR and insulin does not reflect a causal relationship. Nevertheless, an adaptive decrease in myostatin secretion in disadvantageous metabolic conditions counteracting IR would also explain the results. In that case, a parallel to a somewhat similar proposed phenomenon of a decrease in myostatin secretion in conditions strongly predisposing to muscle loss would be appropriate (79).

## Limitations

This study has several limitations. First, a small sample size, resulting from the rarity of the disease, determines low statistical power. Second, the reliability of the measurement of irisin with ELISA is debated as antibodies used in this method may lack specificity (80–82). Third, circulating myostatin represents a mainly latent form composed of C-terminal biologically active peptide non-covalently bound with propeptide, which inhibits its action. This complex is unpaired in target sites. Moreover, myostatin acts principally in a paracrine matter. Considering the above issues, the measurement of serum concentration may not fully correspond to its actual activity.

## Conclusions

Acromegaly is associated with impaired hormonal muscle function characterized by lower irisin secretion independent of the control of the disease. The decrease in irisin secretion may be involved in the development of metabolic complications in this disease. We are first to document the negative association of circulating myostatin levels with GH in acromegaly, which points toward GH as a negative regulator of myostatin secretion; however, this association does not appear to impact muscle mass in this disease significantly.

## Data Availability Statement

The original contributions presented in the study are included in the article/supplementary material. Further inquiries can be directed to the corresponding author.

## Ethics Statement

The studies involving human participants were reviewed and approved by the Bioethics Committee at Wroclaw Medical University. The patients/participants provided their written informed consent to participate in this study.

## Author Contributions

ŁM, MB, and JD designed the project. The first draft of the manuscript was written by ŁM. JH-Ż, MB, and JD wrote, reviewed, and edited the manuscript. Data collection was performed by JH-Ż. KK and AZ performed laboratory measurements and wrote a section of the manuscript. DJ was responsible for body composition measures. JG performed the statistical analysis. All authors contributed to the final version of the manuscript and approved it for publication.

## Funding

This study was supported by grant number SUB.C120.20.016 (Ministry of Science and Higher Education).

## Conflict of Interest

The authors declare that the research was conducted in the absence of any commercial or financial relationships that could be construed as a potential conflict of interest.

## Publisher’s Note

All claims expressed in this article are solely those of the authors and do not necessarily represent those of their affiliated organizations, or those of the publisher, the editors and the reviewers. Any product that may be evaluated in this article, or claim that may be made by its manufacturer, is not guaranteed or endorsed by the publisher.
